# Towards mitigating ecological degradation in G-7 countries: accounting for economic effect dynamics, renewable energy consumption, and innovation

**DOI:** 10.1016/j.heliyon.2021.e08592

**Published:** 2021-12-13

**Authors:** Ojonugwa Usman, Paul Terhemba Iorember, Gylych Jelilov, Abdurrahman Isik, George N. Ike, Samuel Asumadu Sarkodie

**Affiliations:** a School of Business Education, Federal College of Education (Technical) Potiskum, Yobe State, Nigeria; b Department of Economics, Nile University of Nigeria, Nigeria; c Department of Economics, Girne American University, Girne, North Cyprus, Via Mersin 10, Turkey; d Nord University Business School (HHN), Post Box 1490, 8049 Bodø, Norway

**Keywords:** Ecological degradation, Sustainable development, Renewable energy, Renewable research innovations, Economic effect dynamics

## Abstract

The 21^st^ century economic growth is characterized by extensive production and consumption, which increases anthropogenic emissions. However, reducing emission levels require ecological sustainability through innovation and modern technological consideration. This paper investigated not only renewable energy-driven environmental quality but also captured innovation research investment in renewables within the framework of the environmental Kuznets curve (EKC) model for G-7 countries. The findings confirmed the presence of EKC hypothesis for G-7 countries. In addition, renewable energy and innovation were identified to exert negative effects on ecological footprint. To capture the entire conditional distribution of the ecological footprint, we applied the Method of Moments Quantile Regression with fixed-effects. The results affirmed the negative effects of renewable energy innovation. Besides, their effects were heterogeneous across the quantiles with evidence of diminishing effects from lower to higher quantiles, suggesting that countries with lower levels of ecological footprint are possibly more prone to the environmental deterioration effect of income growth. The results of the causality test support economic growth-induced ecological degradation, growth-induced renewables, and innovation-induced ecological conservation. The results further showed a feedback effect between renewables and ecological footprint, innovation, and income growth as well as innovation and renewables. These findings portend important implications for the realization of carbon-free economies in G-7 countries by 2100.

## 1 Introduction

There is a global consensus on the long-term effect of energy consumption, typically fossil fuels on anthropogenic greenhouse gases (GHGs) that hamper environmental quality (Al-Mulali et al., 2015; Charfeddine, 2017; Shujah-ur-Rahman et al., 2019; Asongu et al., 2019; Sarkodie and Strezov 2019; Paramati et al., 2020; Iorember et al., 2020; Usman et al., 2020a & b; Sadik-Zada and Loewenstein, 2020). This phenomenon otherwise known as the “greenhouse effect” has led researchers and policymakers in energy and environmental affairs to explore various avenues that could facilitate a decline in GHGs and curb the menace of ecological degradation vis-à-vis global climate variability. One of the earliest efforts in this regard was the 1992 UNFCCC^1^ with the directive of alleviating the levels of atmospheric GHG concentrations that is not harmful to food production and ensuring sustainable development. Moreover, the Kyoto Protocol Agreement in 1997, the Doha Agreement in 2012, the Paris agreement (COP 21) in 2015, the Sustainable Development Goals (SGDs) in 2016, and other stakeholder engagements all suggest that the current trends in energy consumption is unsustainable and should be reversed. Consequently, many countries including the biggest consumers of fossil fuels such as the US, Germany, China, France, Sweden, Norway, and Italy have adopted decarbonization strategies that ensure a smooth transition to the use of renewables – which have low levels of carbon content (Kannan and Marappan, 2011; Timmons et al., 2014; International Energy Agency, 2015; Iorember et al., 2020; Ike et al., 2020a; Usman et al., 2021a).

It is noteworthy that the transition to renewables is motivated by certain factors, including environmental degradation concerns (particularly, global warming and climate change resulting from excessive carbon emissions), depleting nature of fossil fuels, and uncertainties in oil prices (Owusu and Asumadu, 2016). The substitution of fossil fuel-based power generation with renewables, clean energy, and less carbon-intensive fuel sources is a major decarbonization strategy (IPCC, 2011; Ike et al., 2020b). This entails discouraging the utilization of energy from fossil or non-renewable sources on the one hand and encouraging or increasing consumption of energy from renewable sources with the view of ensuring environmental sustainability on the other hand. Therefore, a rise in the share of renewables in the energy mix is critical to achieving not just a free carbon environment but also a sustainable energy economy. To this effect, the proportion of renewable energy profile is expected to rise in 5 years to achieve a growth of 12.4% by 2023—constituting 30% of the total energy mix, and about 70% of worldwide electricity production growth from renewables. Hence, renewables are anticipated to grow faster and form a greater share of the energy mix than other energy technologies by 2023 (REN21, 2019; IEA, 2019; Ike et al., 2020a; Usman et al., 2020d; Rafindadi and Usman 2020). The variety of energy sources in the energy consumption matrix also confers other gains. For instance, energy utilization from renewables can guarantee energy supplies security as well as solve the problem of local ecological degradation, especially in the G-7-bloc, which forms the focus of our study due to the low environmental damaging content of renewable energy coupled with the stringent environmental laws and commitments to combating pollution. The rise of renewables might also be a source of employment creation especially in areas of “green” technologies and related ancillary economic activities that ensure green growth (Paramati et al., 2020; Balcilar et al., 2018, 2019; Sadik-Zada and Ferrari, 2020). Despite the potential economic and environmental benefits of renewable energy use, the existing evidence suggests that the contribution of renewable sources in global energy utilization is quite low. Based on the World Bank report in 2020, renewables make up 18% of the overall global energy utilization in 2015 and less than 20% in most of the G7 countries.

To achieve a sustained rise in the proportion of renewables to total energy use in the energy mix as well as ensuring green growth (i.e., growth without compromising the environment), there is a need for sustainable investment in renewables via research & development. G-7 countries can enhance their deployment of renewable energy through renewable energy research and innovations. Aside from the fact that energy research innovations can enhance economic growth (Shahbaz et al., 2018; Zafar et al., 2019; Paramati et al., 2020), it can also improve the competitiveness of renewable energy technologies through a reduction in production costs and time. This leads to making the deployment of renewable energy more effective and efficient. Similarly, innovation connected with renewables may have a strong influence on renewable energy and, thus, carbon emissions—by promoting energy efficiency and environmental quality (Balsalobre-Lorente et al., 2018). Technological innovations, especially in energy are very fundamental in the transition to sustainable energy use such as renewables (Shahbaz et al., 2018). This is also noted by Aldieri et al. (2020) that technological innovation promotes firm's knowledge sourcing, which can enhance environmental sustainability. However, Ike et al. (2020a) observe that the deployment of renewable energy and clean energy technologies requires huge investment in energy research and innovation, which is lacking in the current scenario globally. For instance, investment in renewable energy research and innovation has remained insufficient in the global context, amounting to about $17 billion as of 2014 (IEA, 2015).

However, the consumption of renewables, as well as research and development expenditures have gained policy attention across the G-7 countries over the years. This suggests considerably that the growth of these variables could help to mitigate the level of ecological degradation in these countries. Therefore, the current study seeks to provide a more robust perspective on the environmental benefits of renewable energy utilization and investment in energy research innovations. That is, if policymakers understand in clear terms (supported by empirical results) how a unit of investment in renewable energy research and innovation contributes to reducing the ecological footprint and improving environmental quality, the better they are in making informed decisions that can reduce global warming and climate change. Given the important role of technological innovations in defining the global idiosyncrasies in renewables and global climate change mitigation through investment in cleaner energy technologies (Aldieri et al., 2020; Sadik-Zada and Ferrari, 2020), this study primarily investigates the effect of renewable energy utilization and innovation on ecological degradation in G-7 countries. Besides, the study examines the impact of widespread wealth on ecological sustainability within the framework of the EKC hypothesis. As inspiring as studies for G-7 countries may seem, our tour of the literature reveals no such empirical study exists for G-7 countries even though these countries consume more renewables and as such, promote policies for alternative uses of energy.

Studies on the growth–energy-environment nexus have mostly employed carbon dioxide emissions (CO_2_) to measure environmental/ecological degradation, even though CO_2_ emissions represent only an aspect of environmental quality – air pollution (see Shahbaz et 2016; Ulucak and Lin, 2017; Shahbaz et al., 2019; Usman et al., 2019; Yilanci et al., 2019; Usman et al., 2020a; Saud et al., 2020; Iorember et al., 2020; Usman et al., 2021b). To produce more robust and efficient results and track the impact of climate change policy, we employ a more holistic measure of environmental quality termed the ecological footprint—which accounts for the entire biosphere. In other words, the ecological indicator is a useful indicator for assessing natural resource demands and environmental sustainability (Ulucak and Lin, 2017; Sarkodie, 2020, 2021). As noted by Rudolph and Figge (2017), the variable termed the ecological footprint is an aggregate indicator determined based on six sub-components of biodiversity land-use types, namely crop, foraging, forestry, fishing grounds, built-up land, and carbon footprint. The ecological footprint index captures anthropogenic modifications to the entirety of the natural environment.

While previous empirical studies (Alola et al., 2019a; Alola et al., 2019b; Iorember et al., 2021; Ike et al., 2020a; Usman et al., 2020a & b; Bekun et al., 2019; Usman et al., 2020c; Musa et al., 2021) have investigated the extent to which renewable energy consumption aid in mitigating climate change by way of reducing CO_2_ emissions, others have evaluated the link between total energy research innovation and environmental quality (Balsalobre-Lorente et al., 2018). Thus, the present study contributes to the existing literature in four ways: First, the study jointly examines the impact of renewable energy research and innovations and renewable energy on the ecological space. Second, we employ a more comprehensive measure of ecological degradation, viz. the ecological footprint. Many recent studies have used the ecological footprint instead of CO_2_ to capture ecological degradation (Solarin and Bello 2018; Shujah-Ur-Rahman et al., 2019; Destek and Sarkodie 2019; Dogan et al., 2019; Danish et al., 2019; Yilanci et al., 2019; Baloch et al., 2019), but the focus of their studies excluded energy research innovation. Third, this study departs from the previous econometric techniques (i.e., Pooled Mean Group (PMG) – Autoregressive Distributed Lag (ARDL)) usually employed in the literature. Particularly, we use recently developed quantile panel regression via method of moments quantile regression (MMQR) with fixed-effects and robust standard errors by Machado and Silva (2019). Using this technique, we capture the conditional mean which is only located at the center of the distribution (as in the case of PMG/ARDL), and also make use of all information about the points in the entire conditional distribution (as in the case of MMQR). The MMQR model is robust to heterogeneity associated with the panel of countries, hence it is more superior to other versions of panel quantile regressions. Besides, we employ the bias-corrected and accelerated bootstrap confidence intervals for the MMQR estimator to control for cross-sectional dependence. Fourth, to understand the likely causal relationship between the variables, we explore the panel Granger causality with heterogeneous inputs.

The remainder of this study is organized as follows: Section 2 reviews previous empirical studies whereas Section 3 outlines the data and methods employed. Section 4 presents empirical results and discussion while Section 5 concludes the paper with policy implications.

## 2 Previous empirical studies

A few empirical studies have examined the link between research and development and environmental degradation (CO_2_ emission) in both specific and multiple countries using different estimation techniques (Yang et al., 2014; Fernandez et al., 2018; Churchill et al., 2019; Alam et al., 2019; Huang et al., 2020; Paramati et al., 2020). The findings reveal that research and development contribute to the mitigation of environmental degradation. For example, Fernandez et al. (2018) analyzed the link between research and development on CO_2_ emissions for the 15 EU countries including China and the USA from 1990 and 2013. The study finds that investment in research and development effectively leads to a reduction in CO_2_ emissions. Employing the recently developed SOR unit root test and bootstrap bounds cointegration test, Shahbaz et al. (2018) reveal the negative role of energy research innovations on CO_2_ emissions in France. Churchill et al. (2019) examined the association between research and development and CO_2_ in G-7 countries from 1870-2014 using the non-parametric panel technique. The effect of research and development on CO_2_ emissions is characterized by time variance with negative effects for three-quarters of the study. Similarly, Alam et al. (2019) analyzed the effect of corporate research and development on firm environmental performance for G-6 countries. Exploring robust econometric techniques on time series data from 2004 to 2016, the outcomes suggest carbon emissions decline significantly with an increase in research and development.

In another study, Huang et al. (2020) investigated the relationship between research and development and carbon emissions in China using a unique panel dataset for a short period spanning 2000 to 2016. The results show research and development reduce CO_2_ emission intensity. Applying the heterogeneous panel regression and FMOLS, Paramati et al. (2020) examined the linkages between research and development investment on renewable energy consumption and environmental sustainability (captured by CO_2_ emissions) for 25 EU countries. The results suggest that investment in research and development enhances not only renewable energy consumption but reduces CO_2_ emissions. Conversely, Petrovic and Lobanov (2020) examined the impact of research innovations spending on carbon emissions for 16 OECD blocs from 1981 to 2014 using heterogeneous panel regression. The findings show a mixture of positive and negative effects across countries. Equally, the studies of Kocak and Ulucak (2019) and Sagar and Van der Zwaan (2006) could not establish a significant link between renewable energy research and innovation and environmental degradation as well as between total energy innovations and energy intensity, respectively.

In addition, Solarin and Bello (2018) used a STIRPAT model to examine the effect of the total energy innovation on environmental quality in the US. The results indicate that energy innovation has a positive and significant effect on environmental quality. Yet, Usman et al. (2021c) reveal renewable energy innovations significantly decline CO_2_ emissions in the US but such a result is not evident when the ecological footprint is used as a proxy for environmental indicator.

Regarding the energy-growth-environment nexus, several studies (Al-Mulali et al., 2015; Boluk and Mert, 2015; Pata, 2018; Sharif et al., 2019; Ike et al., 2020a & c; Iorember et al., 2020) have specifically accounted for renewables in exploring the link between growth and environmental degradation in the context of the EKC hypothesis. Their findings confirm that renewable energy use contributes to improvement in environmental quality. Similarly, other studies (Dogan and Seker, 2016; Dogan and Ozturk, 2017; Zoundi, 2017; Sinha et al., 2017; Alvarez-Herranz et al., 2017; Balsalobre-Lorente et al., 2018; Allard et al., 2018; Wang and Dong, 2019; Zafar et al., 2019; Usman et al., 2020 a & b) in this regard find evidence to support the contribution of renewables in reducing environmental deterioration.

In summary, the review of the extant literature suggests that several studies explore the effect of research and development on environmental deterioration in single or multiple countries using CO_2_ as a measure of environmental degradation. However, none of the empirical studies examined the influence of research and innovation on environmental degradation using a more robust and comprehensive measure of environmental deterioration; the ecological footprint and particularly focusing on the G-7 countries. Besides, to the best of our knowledge, the existing studies are based on conditional mean estimations, which are invariably located at the center of the distribution, thereby giving an incomplete description of the distributional dynamics. Therefore, the present study fills these research gaps by using renewable energy research and innovation data rather than total research and development data as commonly used in literature to precisely determine the environmental importance of renewable energy-specific innovations. Our study also uses the ecological footprint rather than carbon emissions as a measure of ecological degradation and particularly focuses on the G-7 countries. Finally, we explore the recently developed MMQR with fixed effects, which makes use of the entire conditional ecological distribution function. To this end, the present study is significantly different from the extant literature by way of purpose, data, scope, and methodology.

## 3 Data and methodology

### 3.1 Data

Our study makes use of a balanced panel data comprising ecological footprint per capita, income (lnGDP), second-degree polynomial of income (lnGDP^2^), and Investment in renewable energy R&D per capita. These variables, their measurements, and sources are shown in Table 1.

**Table 1:** Variable, measurement, and source.

Variable	Measurement	Source
Ecological footprint	Global hectares per person	Global Footprint Network (GFN)
Economic Growth (GDP)	Real Gross Domestic Product (GDP) per capita (Constant, 2010 USD).	World Development Indicators
Renewable Energy (RE)	Share of renewables to total primary energy supply in Thousand toe (tonne of oil equivalent)	OECD
Investment in Renewable Energy R&D(REI)	Research Design and Development (RD&D) expenditure in renewable energy technologies (constant 2019 US Dollars and exchange rates)	IEA

### 3.2 Empirical model

**Figure 1:**
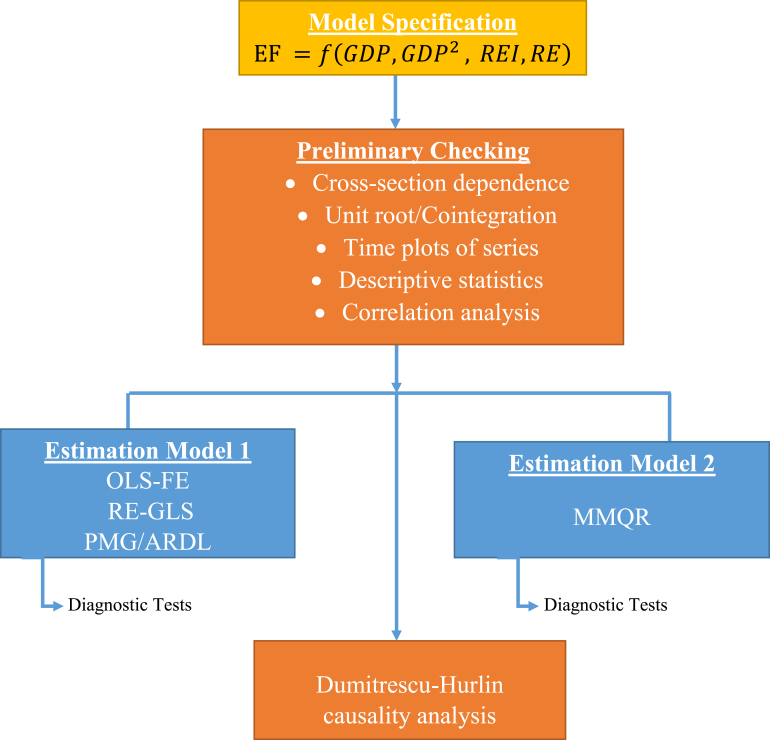
The flow chart of the estimation procedures.

The flow chart in Figure 1 shows the step-by-step empirical techniques used in this study. It begins with the model specification followed by the preliminary checks of the data series, and the estimation of both conditional mean regression models and panel quantile regression model via MMQR. The appropriate diagnostic tests for the models are performed while the last stage of the estimation procedures employs causality analysis. Following the objective of this paper as earlier stated, the empirical model may be expressed as:

(1) $$\text{EF ​}=f\left(\mathrm{GDP},{\mathrm{GDP}}^{2}\text{, ​}REI,RE\right)$$

where EF accounts for environmental degradation, GDP measures the level of income, lnGDP^2^ measures the second-degree polynomial of income, RE represents renewable energy, while REI is the level of Research Design and Development (RD&D) expenditure in renewable energy technologies measured at constant 2019 US Dollars and exchange rates. In a nutshell, Eq. (1) would explain how environmental degradation is determined by the changes in GDP and its squared, renewable energy, and the level of spending on renewable-based R&D. The logarithmic transformation of model 1, is represented by the following function:

(2) $$\ln\;EF_{it}=\text{ ​}\Phi_{0}+\text{ ​}{ф}_{1}\;\ln\;GDP_{it}+\text{ ​}{ф}_{2}\;\ln\;GDP_{it}^{2}+\text{ ​}{ф}_{3}REI_{it}+\text{ ​}{ф}_{4}\;\ln\;RE_{it}+\varepsilon_{it}$$

where $\Phi_{0}$ is the constant term, lnEF is per capita ecological footprint transformed to its natural logarithm, and lnREI is Research, Design, and Development (RD&D) expenditure on renewable energy technologies transformed to natural logarithms. ε is the white noise characterized by stochastic and normal distribution assumptions with zero mean, while the cross-section dimension is represented by *i* (i = 1, 2, …,7) and year period *t* (t = 1985, 1986, …, 2016). Real GDP which relies on investments and transactions in all segments of the economy is expected to put pressure on the ecological space and is thus expected to positively affect the ecological footprint. Renewables and research innovation are expected to improve the ecological space by enhancing the use of energy sources that are not ecologically degrading. To control for cross-country correlation and auto-correlation, the fixed effects OLS (FE-OLS) and the random effects GLS (RE-GLS) regressions, which are augmented with Driscoll-Kraay (DK) standard errors are employed based on Eq. (2).

#### 3.2.1 PMG/ARDL technique

In this study, we first apply the PMG/ARDL technique advanced by Pesaran and Smith (1999). This technique unravels the estimates of the ecological footprint function in both the long- and short-run, expressed as:

(3) $$\ln\;EF_{it}=\;\psi_{i}+\;\sum_{k=1}^{p}\varphi_{ik}\;\ln\;EF_{it-k}+\sum_{k=0}^{q}\theta_{ik}X_{it-k}+\;\varepsilon_{it}$$

where the parameter $\psi_{i}$ is cross-sectional effects while both $\varphi_{ik}$ and $\theta_{ik}$ are the unknown estimated parameters. Hence;

(4) $$\Delta \;\ln\;EF_{it}=\;{ф}_{i}\;\ln\;EF_{it-1}-\sigma_{i}X_{it}+\sum_{k=1}^{p-1}\vartheta_{ik}\Delta \;\ln\;EF_{it-k}+\sum_{k=0}^{q-1}\rho_{ik}\Delta X_{it-k}+\;\varepsilon_{it}$$

where the adjustment parameter/speed of convergence $ECM\left(\vartheta_{i}\right)$ and the long-run coefficients $\left(\sigma_{i}\right)$ are estimated from the first part of the expression ${ф}_{i}\;\ln\;EF_{it-1}-\sigma_{i}X_{it}$ of Eq. (4) while the second part in the right-hand side displays the estimates of the short-run. *X* indicates the vector of the explanatory variables.

#### 3.2.2 Machado-Silva MMQR technique

To capture the effect of all the explanatory variables on ecological footprint in the entire distribution, we apply the panel MMQR as expressed by Machado and Silva (2019) as follows:

(5) $$QEF_{it}=\alpha_{i}+X_{it}^{\prime }\beta +\left(\delta_{i}+Z{΄}_{it}\gamma \right)\varepsilon_{it}$$

Here, the unknown parameters are represented by ${\left(\alpha ,\;\beta^{\prime },\;\delta ,\;\gamma^{\prime }\right)}^{\prime }$, $\;\left(\alpha_{i},\delta_{i}\right),i=1,\ldots ,n$ is the country-specific *i* fixed-effects and $Z^{\prime }$ is the *k-*vector of known, which are the differentiable transformations of the components of *X*_*it*_ with element *l* given by $Z_{l}=Z_{l}\left(X_{it}\right)$ where l=1,…,k. The probability $\text{P}\left\{\delta_{i}+Z_{it}^{\prime }\gamma >0\right\}=1,$ and $\varepsilon_{it}$ represents the random variables that are independent of the strictly exogenous sequence, *X*_*it*_. Eq. (5), therefore implies that:

(6) $$QEF\left(\tau |X_{it}\right)=\left(\alpha_{i}+\delta_{i}q\left(\tau \right)\right)+X_{it}^{\prime }\beta +Z_{it}^{\prime }\gamma q\left(\tau \right)$$

Following Machado and Silva (2019), $q\left(\tau \right)=F_{U}^{-1}\left(\tau \right)$ and hence P(U<q(τ))=τ.

#### 3.2.3 Dumitrescu-Hurlin (D-H) causality analysis

After we have examined the equilibrium nexus between the variables, we utilize the panel Granger causality test to ascertain their causal linkages. The test is robust to heterogeneity in panel data and is suitable even though the time dimension of the panel is larger compared to the cross-sectional dimension as displayed in our case or vice versa (Dumitrescu and Hurlin, 2012). Besides, it can be performed with a bootstrap procedure to mitigate the effects of cross-sectional dependence (C-D).

## 4 Empirical analysis and discussions

### 4.1 Statistical analysis

**Figure 2:**
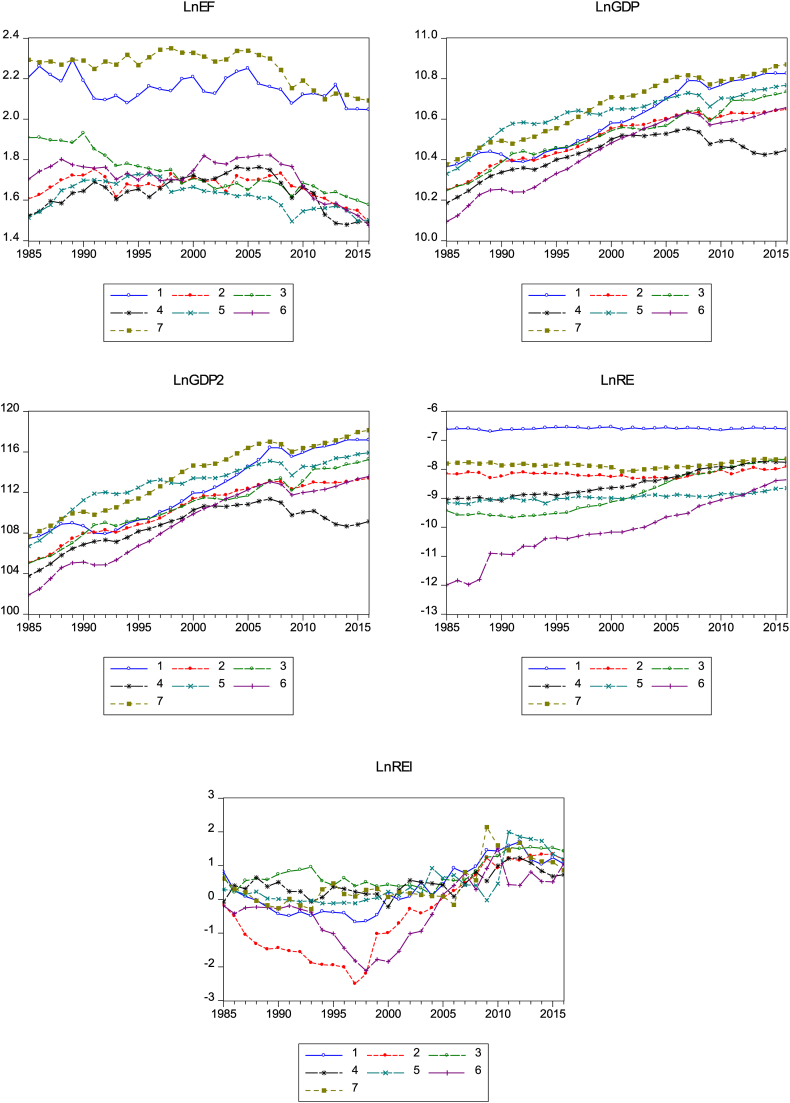
Graphical representation of variables for all countries in the panel.

The descriptive statistics of the panel data explored are presented in Table 2. According to Table 2, the quadratic term of log GDP has the largest mean score. This is followed by the log of GDP and log of ecological footprint. The mean scores of the log of renewable energy and energy technology research design and development expenditures are negative. Table 2 further shows that apart from the standard deviation for the log of GDP squared, the values of the standard deviation for all the remaining variables are within the range of zero, which suggests that the values of these variables are not volatile except the quadratic GDP term. Furthermore, while the log of ecological footprint displays a positive skewness, the rest of the variables have negative skewness. The kurtosis of the variables is all positive with the log of renewable energy and energy technology research design and development expenditures suggesting excess kurtosis. Consequently, the Jarque-Bera statistics for the log of ecological footprint, renewable energy, and energy technology research, design, and development expenditure reject the range for normal distribution. This implies that a bell shape is not established for ecological footprint, renewable energy, and energy technology research, design, and development expenditure. The correlation coefficient matrix of variables in Table 3 shows that the correlation between variables is not too high, which perhaps suggests no evidence of likely multicollinearity among the variables. The correlation between all the variables in their logs exhibits positive significance except that between the log of the ecological footprint and energy technology research, design, and development expenditures, which is negative. From Figure 2, it can be observed that all the variables follow almost the same evolutionary dynamics in G7 countries with Canada and the USA (lines 1 and 7) having a greater ecological footprint amongst countries in the G7, which may not be unconnected to their high dependence on natural resources extraction, specifically—crude oil extraction. It can also be observed that the ecological footprint of all G7 countries elicits diminishing dynamics over time due to awareness of the risks in climate change and the need to initiate mitigation policies. The economic growth of this set of countries also follows a tightly knit evolution through time. Consumption of renewable energy and innovations in renewable energy is however a bit more disparate. This underscores the different country-specific government and stakeholder attitudes concerning renewable energy utilization and renewable energy innovations.

**Table 2:** Descriptive statistics.

Variable	LNEF	LNGDP	LNGDP2	LNREI	LNRE
Mean	1.826	10.539	111.099	-13.566	-8.420
Median	1.719	10.550	111.300	-13.512	-8.293
Maximum	2.349	10.870	118.149	-11.685	-6.534
Minimum	1.475	10.095	101.903	-16.315	-11.988
Std. Dev.	0.257	0.163	3.437	0.860	1.117
Skewness	0.754	-0.183	-0.149	-0.701	-0.421
Kurtosis	2.114	2.489	2.464	3.765	3.677
Jarque-Bera	28.540	3.687	3.510	23.776	10.883
Probability	0.000	0.158	0.173	0.000	0.004
Observations	224	224	224	224	224

**Table 3:** Correlation matrix analysis.

LNEF	1				
	–				
LNGDP	0.224	1			
	0.001	–			
LNGDP2	0.225	1.000	1		
	0.001	0.000	–		
LNREI	-0.059	0.500	0.502	1	
	0.931	0.0000	0.0000	–	
LNRE	0.504	0.463	0.461	0.237	1
	0.000	0.000	0.000	0.000	–

### 4.2 Pesaran test for cross-sectional dependence (CD)

Having examined the descriptive statistics of the series employed in this study, the next stage is to test for cross-sectional dependence. In doing this, we apply a cross-sectional dependence test developed by Pesaran (2021). Evidence from Table 4 indicates that the null hypothesis of no cross-sectional dependence is rejected at *p-value < 0.01*. This implies that there is cross-sectional dependence in the analyzed variables across the G-7 countries.

**Table 4:** Pesaran test for cross-sectional dependence.

Variable	C-D Test	*p*-value	*Corr.*	Abs(Corr)
LNEF	13.81∗∗∗	0.000	0.533	0.533
LNGDP	23.88∗∗∗	0.000	0.921	0.921
LNREI	18.55∗∗∗	0.000	0.716	0.716
LNRE	11.62∗∗∗	0.000	0.448	0.468

Note: ∗∗∗ represents the rejection of the null hypothesis (i.e., there is cross-sectional independence across countries in the panel) at 1% significance level.

### 4.3 Panel unit root test results

Table 5 displays the results of the stationarity tests based on the IM-Pesaran-Shin tests and that of CIPS tests advanced by Pesaran (2007). While IM-Pesaran-Shin accounts for heterogeneity, Pesaran CIPS accounts for C-D and heterogeneity across the variables. The results of these tests reveal that under IM-Pesaran-Shin, lnGDP and its squared term as well as lnRE are mean-reverting in level and first difference while lnEF and lnREI are first difference stationary. While controlling for variable cross-section dependence and heterogeneity, the results from the CIPS panel unit root test show all variables are I(1), with the implication of non-stationarity at levels and stationarity at first-differences.

**Table 5:** Panel unit-root tests.

Variable	Constant	Constant & trend	Constant	Constant & trend
	At Levels		First Difference	
**IM-Pesaran-Shin Unit Root Tests**				

lnEF	-1.950	-0.996	-8.312∗∗∗	-8.852∗∗∗
lnGDP	-2.158∗∗	-0.188	-5.234∗∗∗	-5.944∗∗∗
${\mathrm{lnGDP}}^{2}$	-1.980∗∗	-0.194	-5.285∗∗∗	-5.970∗∗∗
lnREI	-1.051	-2.903∗∗∗	-8.510∗∗∗	-8.562∗∗∗
lnRE	-2.863∗∗∗	-2.443∗∗	-8.322∗∗∗	-8.430∗∗∗
**CIPS Panel Unit Root Tests**				

lnEF	-2.715∗∗∗	-2.787∗∗∗	-5.453∗∗∗	-5.544∗∗∗
lnGDP	-1.341	-1.771	-2.985∗∗∗	-2.873∗∗∗
${\mathrm{lnGDP}}^{2}$	-1.329	-1.748	-2.980∗∗∗	-2.873∗∗∗
lnREI	-3.064	-3.087	-5.688∗∗∗	-5.792∗∗∗
lnRE	-2.043	-2.616	-5.225∗∗∗	-5.433∗∗∗

Note: ∗∗∗ and ∗∗ indicate rejection of the null hypothesis at 1% and 5% significance level respectively.

### 4.4 Cointegration analysis results

To test for the long-run nexus (cointegration) among variables, we employ a test developed by Westerlund (2007). Since some series employed in this study are not normally distributed and also to mitigate the potential effects of C-D, the bootstrap simulations version of Westerlund's tests is applied to ensure reliable critical values when cointegration tests are conducted. Westerlund's cointegration tests also control for the effects of cross-country correlation as well as heterogeneity. The results of these tests as displayed in Table 6 show that two (2) of the four specifications of the test reject the null hypothesis of no cointegration when Westerlund's cointegration tests are conducted with asymptotic standard errors. Moreover, the results of the bootstrap simulations version of the tests also indicate that two (2) of the four specifications give valid support for cointegration. By implication, cointegration is validated among the variables explored in this study.

**Table 6:** Westerlund Panel cointegration tests.

Statistic	Value	Z-value	*p*-value	Robust *p*-value
Gt	-3.130∗∗∗	-2.962	0.002	0.000
Ga	-7.729	0.800	0.788	0.270
Pt	-7.762∗∗∗	-2.751	0.003	0.000
Pa	-7.413	-0.470	0.319	0.150

Note: The tests are conducted with a maximum lag of zero whereas ∗∗∗ represents the rejection of the null hypothesis at 1% significance level.

### 4.5 OLS-FE, RE-GLS, and PMG/ARDL results

Having established evidence of integration and cointegration amongst the variables, we step further to estimating models explored in this study. First, the OLS-FE, RE-GLS, and PMG/ARDL models are explored. The results of the coefficients for PMG/ARDL are presented in Table 7 for both long run and short run. The growth of the economy has an increasing effect on the ecological footprint while the squared term of economic growth reduces the pressure on the ecological space. To curtail the possible effect of the C-D and autocorrelation, we estimate the model with OLS-FE and RE-GLS methods. The EKC hypothesis is thus validated for all 3 employed mean-based estimators. The result implies that at the lower levels of income, environmental challenges are associated with an increase in income level but at the higher levels of income, increasing income level would reduce environmental degradation. This is traceable to the awareness of the necessity of environmental protection and the ability of the government to shoulder the investment cost burden of switching fuels at this income level. The implication for the validation of the EKC hypothesis is that––the quest for economic development escalates ecological degradation during the formative years, however, as economic development reaches a certain threshold, the challenges of ecological degradation declines. Therefore, this finding agrees with Ike et al. (2020a), which reveals the EKC for G-7 countries in both the panel and country-specific analyses to be inverted U-shaped. Moreover, a 1% surge in research innovation would reduce EF (ecological footprint) by 0.013% while a 1% increment in renewable energy would cause EF to diminish by about 0.49%. This finding echoes the empirical finding of Dogan and Seker (2016), Dogan and Ozturk (2017), Zafar et al. (2019). The finding, however, disagrees with Sadik-Zada and Loewenstein (2020), which strongly rejects an inverted income-emission nexus and establishes a positive income-emission linkage for oil-producing countries with evidence of over-proportionate increase in per capita CO_2_ emissions following a rise in income.

**Table 7:** OLS-FE, RE-GLS, and PMG-ARDL coefficients.

Variable	PMG/ARDL	OLS-FE	RE-GLS
**Long-run Coefficients**			

lnGDP	27.78∗∗∗ (8.6583)	24.298∗∗∗ (8.413)	20.804∗∗∗ (3.8257)
${\mathrm{lnGDP}}^{2}$	-1.3139∗∗∗ (0.4087)	-1.1512∗∗∗ (0.1621)	-0.9861∗∗∗ (0.1817)
lnREI	-0.0125 (0.0114)	-0.0144∗∗∗ (0.0069)	-0.0179∗∗∗ (0.0079)
lnRE	-0.4924∗∗∗ (0.0796)	-0.1080∗∗∗ (0.1145)	-0.0884∗∗∗ (0.0125)
Constant		-127.47∗∗∗ (17.981)	-108.86∗∗∗ (20.147)
**Short-run Coefficients**			

ECT(−1)	-0.2302∗∗ (0.0822)		
lnGDP	7.9479 (12.107)		
${\mathrm{lnGDP}}^{2}$	-0.3327 (0.5734)		
lnREI	-0.1040∗∗∗ (0.0074)		
lnRE	-0.0804∗∗ (0.0328)		
Constant	-34.2816∗∗ (12.203)		

Note: ∗∗∗, ∗∗ and ∗ indicate rejection of the null hypotheses at 1%, 5%, and 10% significance level, respectively while the maximum lag (i.e., 2) is selected based on AIC lag-selection technique.

The coefficient of renewable energy innovations is however not significant for all mean-based estimators prompting the use of quantile regression to unravel quantile-based relationships across the conditional distribution of the ecological footprint. The insignificance of renewable energy innovations is congenial with Kocak and Ulucak (2019) wherein no significant effect between renewable energy innovations and pollutant emissions could be unraveled. The results obtained may not be unconnected with the use of static equilibrium models as well as the neglect of the conditional distribution of the pollutant emission. The effect of renewable energy innovations may vary depending on the intensity of environmental degradation in a country as well as stakeholders and the governments’ attitude towards ecological/environmental degradation. Also, it may take a while before the effect of investment in innovations could be felt in the broader economy, thus static equilibrium models may not necessarily capture these dynamics. Moving forward, the negative and significant effect of the utilization of renewable energy is consistent with Usman et al. (2020a & c), and Ike et al. (2020a), which documents that renewable energy utilization of course improves the environment disparately across G-7 economies.

The result of the short run for the case of PMG/ARDL model indicates that the adjustment parameter is negative and highly significant, easily passing 5% significance level. The EKC hypothesis is not statistically validated in the short-run. Furthermore, we find that a 1% increase in research innovation and renewables would reduce the ecological footprint by about 0.104% and 0.080% respectively in the short run. This finding concurs with Shahbaz et al. (2018); Fernandez et al. (2018); Churchill et al. (2019); Paramati et al. (2020).

### 4.6 Panel quantile (MMQR) results

The estimations of the traditional mean regressions explored may be biased as they only make use of the conditional mean, which does not capture the full distributional dynamics. The quantile regression technique proposed by Machado and Silva (2019) captures the entire conditional distribution of the ecological footprint. The estimation employs bias-corrected and accelerated bootstrap standard errors to mitigate the potential effect of C-D.

**Figure 3:**
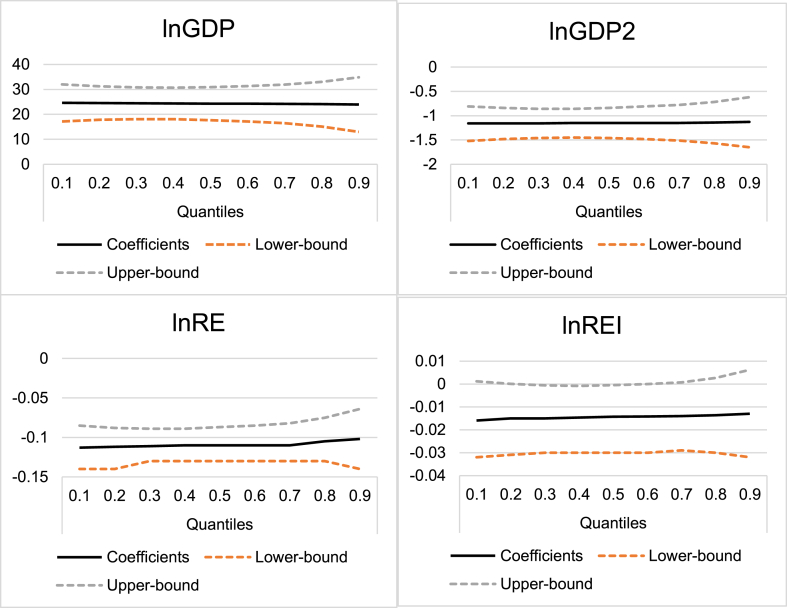
Graphical depiction of quantile coefficients with 95% confidence intervals.

Therefore, Table 8 presents the results of the MMQR regression with the graphical depiction of the results as shown in Figure 3. The location parameters, which are analogous to the Ordinary Least Square-fixed effect (OLS-FE) regression validate the hypothesis of the EKC in the G-7 bloc. The effects of renewable energy consumption and innovation are negative and significant with renewable energy utilization having a greater impact in terms of magnitude. The scale parameters evince evidence showing that the scale of the coefficient effects is not disparate across quantiles except for the coefficient of renewable energy consumption. This may be due to the homogeneous development and technological level of the G-7 economies. What can also be observed from the estimation is that where the traditional mean estimators reject the significance of the renewable energy innovation coefficients, the MMQR estimator however only rejects the statistical significance at the extreme tails of the distribution (quantiles 0.8 and 0.9). Weak statistical significance (0.05 < *p* < 0.1) is observed from quantiles 0.1, 0.2 and 0.7, while strong statistical evidence (*p* < 0.01) is observed from quantile 0.3 up to 0.6. This validates the suitability of the MMQR technique as shown in Ike et al. (2020c).

**Table 8:** Panel quantile estimation results.

Variables	Coefficient	Std. Error	*p*-value
**Location Parameters**			
lnGDP	24.292∗∗	8.433	0.028
${\mathrm{lnGDP}}^{2}$	-1.1508∗∗	0.396	0.027
lnREI	-0.0144	0.014	0.340
lnRE	-0.1080∗∗∗	0.019	0.002
Constant	-127.44∗∗	44.985	0.030
**Scale Parameters**			
lnGDP	-0.1982	3.593	0.958
${\mathrm{lnGDP}}^{2}$	0.0086	0.172	0.962
lnREI	0.0007	0.005	0.883
lnRE	0.0301∗	0.013	0.063
Constant	1.2189	18.79	0.950
**0.1 Quantile Coefficients**			
lnGDP	24.585∗∗∗	6.082	0.000
${\mathrm{lnGDP}}^{2}$	-1.1635∗∗∗	0.288	0.000
lnREI	-0.0155	0.011	0.164
lnRE	-0.1126∗∗∗	0.022	0.000
**0.2 Quantile Coefficients**			
lnGDP	24.516∗∗∗	5.148	0.000
${\mathrm{lnGDP}}^{2}$	-1.1606∗∗∗	0.244	0.000
lnREI	-0.0152∗	0.009	0.093
lnRE	-0.1116∗∗∗	0.019	0.000
**0.3 Quantile Coefficients**			
lnGDP	24.453∗∗∗	4.396	0.000
${\mathrm{lnGDP}}^{2}$	-1.1578∗∗∗	0.208	0.000
lnREI	-0.0150∗	0.008	0.062
lnRE	-0.1105∗∗∗	0.016	0.000
**0.4 Quantile Coefficients**			
lnGDP	24.371∗∗∗	3.674	0.000
${\mathrm{lnGDP}}^{2}$	-1.1543∗∗	0.174	0.000
lnREI	-0.0147∗∗	0.007	0.029
lnRE	-0.1092∗∗∗	0.013	0.000
**0.5 Quantile Coefficients**			
lnGDP	24.277∗∗∗	3.423	0.000
${\mathrm{lnGDP}}^{2}$	-1.1502∗∗∗	0.162	0.000
lnREI	-0.0143∗∗∗	0.006	0.000
lnRE	-0.1077∗∗∗	0.013	0.002
**0.6 Quantile Coefficients**			
lnGDP	24.233∗∗∗	3.559	0.000
${\mathrm{lnGDP}}^{2}$	-1.1483∗∗∗	0.169	0.000
lnREI	-0.0141∗∗	0.007	0.030
lnRE	-0.1071∗∗∗	0.013	0.000
**0.7 Quantile Coefficients**			
lnGDP	24.192∗∗∗	3.822	0.000
${\mathrm{lnGDP}}^{2}$	-1.1465∗∗∗	0.181	0.000
lnREI	-0.0140∗∗	0.007	0.045
lnRE	-0.1064∗∗∗	0.015	0.000
**0.8 Quantile Coefficients**			
lnGDP	24.075∗∗∗	5.055	0.000
${\mathrm{lnGDP}}^{2}$	-1.1415∗∗∗	0.239	0.000
lnREI	-0.0136	0.009	0.143
lnRE	-0.1045∗∗∗	0.019	0.000
**0.9 Quantile Coefficients**			
lnGDP	23.903∗∗∗	7.514	0.001
${\mathrm{lnGDP}}^{2}$	-1.1340∗∗∗	0.356	0.001
lnREI	-0.0129	0.014	0.349
lnRE	-0.1018∗∗∗	0.028	0.000

Note: ∗∗∗, ∗∗ and ∗ indicate the statistical significance at 1%, 5%, and 10% level, respectively.

We find renewable energy research innovation exerts a negative effect on ecological footprint. This infers that spending on renewable energy innovation improves clean energy production and consumption, hence, underpins environmental quality. This is because the environmental degradation effect of renewables is close to non-existent as compared to fossil fuels. This effect is highly significant up to the 70^th^ quantile. The insignificance of renewable energy innovation in the 80^th^ and 90^th^ quantiles could be traceable to the fact that the spending on renewable energy research and development at these higher quantile levels is not enough to mitigate ecological degradation which is at its highest level.

The effects of renewable energy innovation are heterogeneous across the quantiles with strong evidence of diminishing effects from lower to higher quantiles. This result implies that countries with lower levels of ecological footprint are conceivably more prone to the environmental deterioration effect of income growth. This could be because such countries may lower their stringent environmental laws and policies on carbon pricing and taxes on pollutant activities to promote economic growth. Stringent laws and policies may drive away firms and industries to operate where carbon pricing and taxes are minimal thereby causing some economic problems such as high unemployment and inflation as economic activity would dampen significantly. Also, the scenario might be that countries at higher levels of ecological degradation may not prioritize spending more on renewable energy. This empirical finding is consistent with Churchill et al. (2019), who reported that the total R&D innovations reduce carbon emissions in G-7 countries. Our result agrees with Shahbaz et al. (2018) who found similar results for France using the total energy innovations. Furthermore, the result of this study echoes the recent findings of Paramati et al. (2020) who found that the total R&D innovations are environmentally sustainable for EU countries, and Usman et al. (2021c) who confirmed the effect of renewable energy innovation is negative on two measures of environmental degradation (i.e., CO_2_ emissions and ecological footprint) but only statistically significant in the case of CO_2_ emissions.

The effect of renewable energy is negative and significant across the conditional quantile distribution of the ecological footprint. The results further reveal that as renewable energy increases, the level of environmental degradation decreases due to improvement in the efficient use of energy across the quantiles. In other words, renewable energy is more efficient in reducing environmental degradation even in high-income countries where the pressure on the ecological space is close to median levels. This can be seen as the statistical significance of the effect of renewable energy reducing marginally from the lower quantiles to the upper quantiles. This further validates the preference of the MMQR technique over traditional mean-based estimators. These findings from the equilibrium models are close to those documented by Usman et al. (2020a) while the significant reducing effect of renewable energy use agrees with Ike et al. (2020a), Usman et al. (2020b) that renewable energy exerts downward pressure on environmental degradation.

The results from the quantile regression suggest that economic growth effects (income level) are positive and significant while the quadratic term of income is negative and significant across the conditional quantile distribution of the ecological footprint. This confirms the EKC hypothesis. The results from the scale parameters for economic growth suggest that the ecological footprint effect of economic growth may not vary too much across income levels in G-7 countries. The economic reason for this finding is that G-7 countries are all high-income economies at roughly the same stage of economic development, thus it is expected that EKC effects may not be too far apart across these economies. This finding could also be traceable to the awareness of the people about environmental issues when it comes to inducing stringent environmental laws to regulate the pollutant activities of individuals, firms, and industries. Therefore, our findings concur with Usman et al. (2020b).

**Figure 4:**
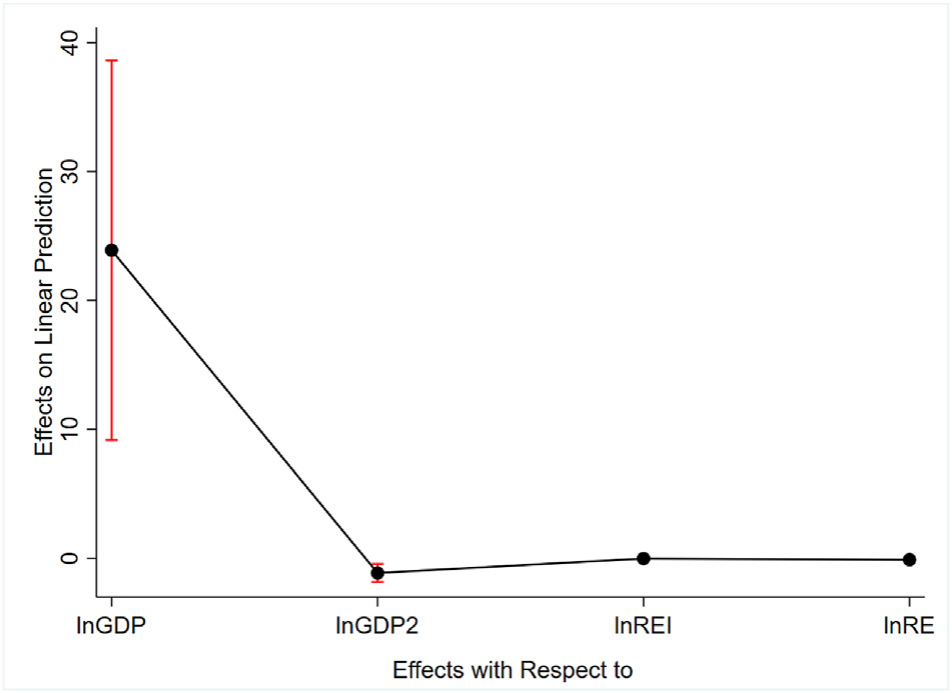
Average Marginal effects with 95% confidence intervals.

Besides, we examined the robustness of the quantile estimation using the average marginal effect based on a 95% confidence interval as shown in Figure 4. The results confirm the validity of the estimated parameters.

### 4.7 Dumitrescu-Hurlin (D-H) causality test results

From a policy perspective, we examined the causal linkage between the variables using the D-H panel causality approach. The application of this panel causality procedure is necessary due to the need to incorporate dynamics in the innovation-environmental degradation nexus and to further understand the direction of the causal effect, which are important for policy formulation––which hitherto cannot be established via equilibrium models. The panel Granger causality procedure employs a bootstrap procedure proposed in D-H (2012) to mitigate the potential effects of C-D. The results as displayed in Table 9, demonstrated that a one-sided causality flows from economic growth to ecological footprint, and from renewable energy innovation to ecological footprint. This validates the utilization of the panel Granger causality procedure as no relationship is unraveled regarding renewable energy innovations and ecological footprint in the traditional mean-based static equilibrium models due to the non-incorporation of the necessary time dynamics (lag-augmentation) needed for the effect of innovations to be fully made manifest in the environment. The results are in line with Usman et al. (2020b) who found a causal flow from economic growth to ecological footprint. Also, Sarkodie (2018) and Rafindadi and Usman (2019) reported a similar result for South Africa and Usman et al. (2019) for India. The causal link between renewables and ecological footprint is two-sided, i.e. it is characterized by a feedback effect. Furthermore, we find a two-sided causality between renewable energy innovation and economic growth and unidirectional causality flowing from renewable energy innovation to renewable energy use. This result concurs with Shahbaz et al. (2018) who find a bidirectional causality between economic growth and spending on energy R&D but contradicts Tsaurai (2017) who establishes that R&D spending only leads to output growth. Finally, the causal link between renewables and income is unidirectional, flowing only from economic growth to renewable energy, which supports the scenario that economic development spurs action towards the deployment of renewable energy sources to maintain environmentally sustainable production processes. This is consistent with Ike et al. (2020a) wherein a unidirectional causality exists, in such a way that economic growth induces renewable energy deployment.

**Table 9:** Dumitrescu-Hurlin causality test results.

Null Hypothesis	Stat.	*P*-value
lnGDP(lnGDP^2^) ≠> lnEF	10.344∗∗∗	0.000
lnEF ≠> lnGDP(lnGDP^2^)	0.336	0.737
lnREI ≠> lnEF	5.185∗∗∗	0.000
lnEF ≠> lnREI	0.449	0.653
lnRE ≠> lnEF	7.471∗∗∗	0.000
lnEF ≠> lnRE	11.170∗∗∗	0.000
lnREI ≠> lnGDP(lnGDP^2^)	3.579∗∗∗	0.000
lnGDP(lnGDP^2^) ≠> lnREI	10.496∗∗∗	0.000
lnRE ≠> lnGDP(lnGDP^2^)	0.953	0.341
lnGDP(lnGDP^2^) ≠> lnRE	4.309∗∗∗	0.000
lnREI ≠> lnRE	2.475∗∗	0.013
lnRE ≠> lnREI	4.800∗∗∗	0.000

Notes: Estimated based on a maximum lag order of 1 whereas ∗∗∗ and ∗∗ represent the statistical significance at 1% and 5^%^ level.

## 5 Conclusion & policy implications

This study examines the different roles of economic growth, spending on renewable energy R&D, and renewables in mitigating the greenhouse effect in G-7 countries over the period 1985–2016. The results of the conditional mean regression estimates via OLS-FE, RE-GLS, and PMG/ARDL models suggest economic growth reduces environmental quality but the impact of the quadratic growth term is negative, which validates the EKC hypothesis for G-7 countries. We also find a rise in renewable energy and investment in renewable energy innovation improves environmental quality. The short-run effects of economic growth and its quadratic term are not statistically significant. Furthermore, the quantile estimation reveals that even though the EKC hypothesis is validated in both lower and upper quantiles, the effect of income across the conditional distribution of the ecological footprint is homogenous in G-7 countries. Our results divulge that the effects of renewable-based research innovation and renewable energy consumption in ameliorating environmental degradation are more efficient at quantiles close to the median. The causality test shows economic growth has predictive content for ecological footprint, thus, renewable energy innovation Granger causes ecological footprint. The causal interactions between renewables and ecological footprint, energy innovation, and economic growth, as well as energy innovation and renewables have a feedback effect. In addition, there is evidence of unidirectional causality from income to renewable energy.

The policy directive of these findings is that economic growth has long-term ecological degradation effect for the sampled countries. Thus, achieving the long-term target of G-7 countries by 2100, viz. a carbon-free economy, requires the decoupling of economic development from environmental degradation via transition from fossil fuels to renewables. To do this, energy policies that stimulate the adoption of renewables (i.e., hydropower, wind, biomass, solar, and geothermal) are needed. Such energy policies could focus on promoting industry 4.0, carbon pricing, taxes, and subsidies on green energy technologies. Besides, adequate funding should be set aside for the development of renewables, R&D, innovation, and technology to enhance not only environmental quality but efficient production and utilization of energy. Irrespective of a country's level of ecological footprint, continuous efforts could be made by stakeholders in government and private sectors to initiate financial support for research and development in clean energy technologies. Even though ecological footprint does not have any predictive content for economic growth, ecological conservation may be a more sustainable route for G-7 countries because it would not alter or diminish economic development. The fact that renewable energy innovation induces economic growth and mitigates ecological degradation whilst having causal interpretations for both equilibrium perspectives make it a viable tool in decoupling economic growth from ecological footprint. Governments and other relevant stakeholders saddled with the responsibility of promoting environmental sustainable could make available grants and subsidies for renewable energy R&D innovations to encourage further investments in this critical sphere of innovation.

Despite the robust findings of this study, it is clear that this study fails to capture the directional asymmetric effect of renewable energy innovations. This is because a positive change in renewable energy innovation may exert a different impact from a negative change of the same magnitude. To this extent, we suggest future studies could capture the effect of directional asymmetry in renewable energy innovation on ecological footprint.

## Declarations

### Author contribution statement

Ojonugwa Usman: Conceived and designed the experiments; Performed the experiments; Analyzed and interpreted the data; Contributed reagents, materials, analysis tools or data.

Paul Terhemba Iorember, Gylych Jelilov & Abdurrahman Isik: Contributed reagents, materials, analysis tools or data; Wrote the paper.

George N. Ike & Samuel Asumadu Sarkodie: Analyzed and interpreted the data; Contributed reagents, materials, analysis tools or data; Wrote the paper.

### Funding statement

This research did not receive any specific grant from funding agencies in the public, commercial, or not-for-profit sectors.

### Data availability statement

Data will be made available on request.

### Declaration of interests statement

The authors declare no conflict of interest.

### Additional information

No additional information is available for this paper.

## Footnotes

1 United Nations Framework Convention on Climate Change.
